# T4SP Database 2.0: An Improved Database for Type IV Secretion Systems in Bacterial Genomes with New Online Analysis Tools

**DOI:** 10.1155/2016/9415459

**Published:** 2016-09-21

**Authors:** Na Han, Weiwen Yu, Yujun Qiang, Wen Zhang

**Affiliations:** ^1^State Key Laboratory for Infectious Disease Prevention and Control, National Institute for Communicable Disease Control and Prevention, Chinese Center for Disease Control and Prevention, Beijing 102206, China; ^2^Collaborative Innovation Center for Diagnosis and Treatment of Infectious Diseases, Hangzhou 310003, China

## Abstract

Type IV secretion system (T4SS) can mediate the passage of macromolecules across cellular membranes and is essential for virulent and genetic material exchange among bacterial species. The Type IV Secretion Project 2.0 (T4SP 2.0) database is an improved and extended version of the platform released in 2013 aimed at assisting with the detection of Type IV secretion systems (T4SS) in bacterial genomes. This advanced version provides users with web server tools for detecting the existence and variations of T4SS genes online. The new interface for the genome browser provides a user-friendly access to the most complete and accurate resource of T4SS gene information (e.g., gene number, name, type, position, sequence, related articles, and quick links to other webs). Currently, this online database includes T4SS information of 5239 bacterial strains.* Conclusions*. T4SS is one of the most versatile secretion systems necessary for the virulence and survival of bacteria and the secretion of protein and/or DNA substrates from a donor to a recipient cell. This database on virB/D genes of the T4SS system will help scientists worldwide to improve their knowledge on secretion systems and also identify potential pathogenic mechanisms of various microbial species.

## 1. Background

Secretion systems, which can mediate the passage of macromolecules across cellular membranes, are essential for virulence and genetic material exchange among bacterial species [1–3]. Type IV secretion systems (T4SS) play various roles in transporting diverse components, from single proteins to protein-protein and protein-DNA complexes [1, 4–7]. Of these, the most important role is their ability to mediate the conjugative transfer of plasmid DNA or transposons, which facilitates the spread of antibiotic-resistant or pathogenic genes among several bacterial species [4, 5, 7, 8]. In addition, T4SS can also direct bacteria to inject genes for encoding themselves into host cells during an infection [7, 9]. Since all T4SS are evolutionarily related [5], they could be identified based on the structures of their genes. However, T4SS in Gram-negative and Gram-positive strains have variable gene numbers and high sequence diversity [8, 10], which limit their identification.

In the last version of T4SP database, 717 experimentally verified* virB/D* genes, 3852 putative functional* virB/D* genes predicted based on genomic location and homology, and 21785 homologs of* virB/D* genes predicted based on homology in 1183 complete bacterial genomes as well as an integrative bioinformatics software package for predicting T4SS in bacterial genomes are publicly available (http://www.secretion.org/) [10]. The Type IV Secretion Project 2.0 (T4SP 2.0) database (http://www.secretion.org/) is an improved and extended version of the platform that currently includes more T4SS related gene data of 5239 bacterial strains. The most advanced web server tools for detecting the existence and variation of T4SS genes online are available in this version. Besides, the new interface for the genome browser provides a user-friendly access to the most complete and accurate resource of T4SS gene information (e.g., gene number, name, type, position, sequence, related articles, and quick links to other webs).

## 2. Construction and Content

In this update, we expanded the database to include 5239 bacterial strains. We downloaded genome sequences of these bacterial strains and used the T4SP analysis package to detect T4SS genes at the genome level. A total of 6191 virB/D genes predicted based on genomic location and homology and 51,067 homologs of virB/D genes, as well as their related information (such as gene sequence, location, direction, and related references), were stored in a T4SP 2.0 database (Figure 5). Users could search the database by the taxonomy tree (Figure 6) in the T4SS database page.

## 3. Utility and Discussion

### 3.1. New Online T4SS Prediction Analysis Tool and Blast Tool

**Figure 1:**
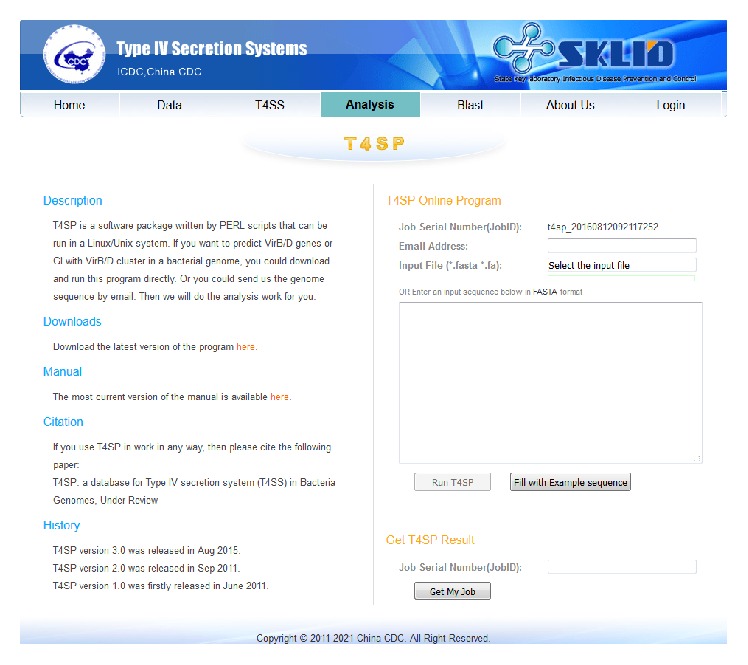
The T4SP analysis page where users could upload their DNA sequences on the web page and get the report page about the T4SS genes.

Since many users are unfamiliar with bioinformatics tools, they could not use the local command tool in our previous version to analyze their data [10]. Hence, we included a new online T4SS prediction analysis tool in this version. Users can simply upload their DNA sequences on the web page (only supporting files in fasta format) and click “Run T4SS” (Figure 1). Then a status page showing the current status of this analysis task is displayed. After usually less than five minutes, the result link will be automatically shown on the status page. The analysis process of how to get this result was described in detail in our previous papers [8, 10]. A graphical report with genome browser and tables displaying all the information of T4SS genes related to this DNA sequence will be shown (Figure 4). Predicted T4SS genes and clusters are marked in the genome with colored masks. In the genome browser, users can also see the detailed gene sequence, start site, end site, type and direction, and gene name by clicking the corresponding mask on the figure. The predicted T4SS regions, which cover several T4SS genes, are also masked in the genome browser and their detailed information can be seen by clicking on the figure or directly in the table shown below. Users can detect the existence of T4SS online and find the distribution of these T4SS genes, which will be useful for further experiments.

**Figure 2:**
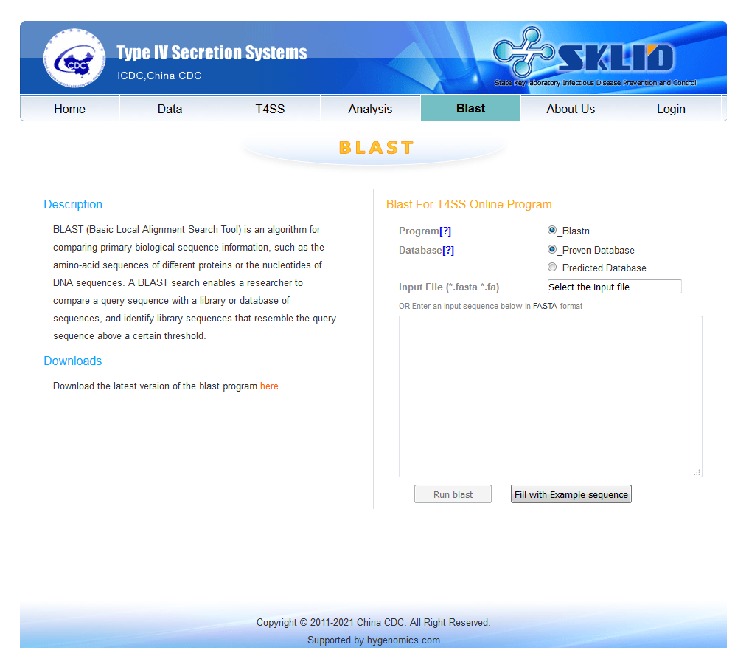
The online blast page.

**Figure 3:**
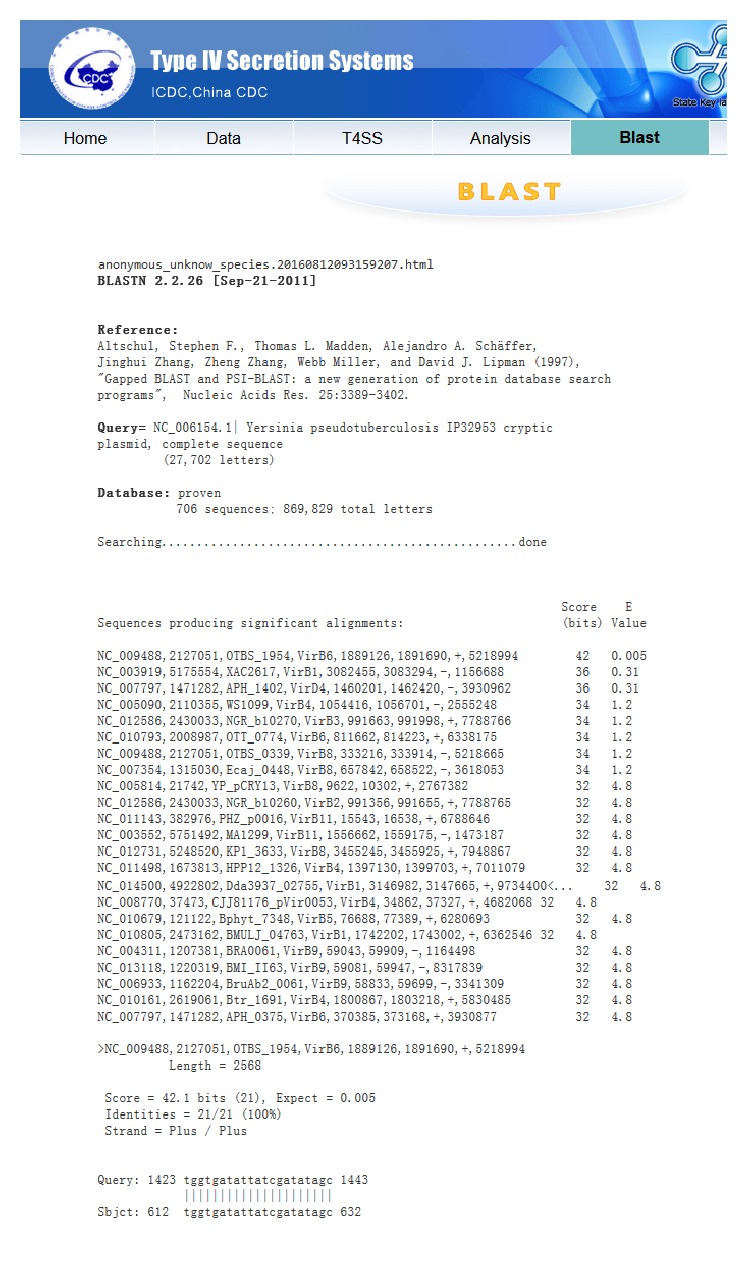
The blast result page.

For comparing nucleotide sequences of T4SS genes, T4SP 2.0 also supports the online blast version (Figure 2). Two databases, proven T4SS genes database and predicted T4SS genes database, are available. Users can select a database, upload or copy and paste their gene sequences, and then click “Run blast.” The results page will be shown in a minute, which lists all matches for the corresponding database. With the help of this tool, users can find the most similar gene sequence and predict the candidate evolution process. The mutation sites in genes can also be distinguished by this analysis tool (Figure 3).

### 3.2. New Version of Gene Browser and Other Functions

**Figure 4:**
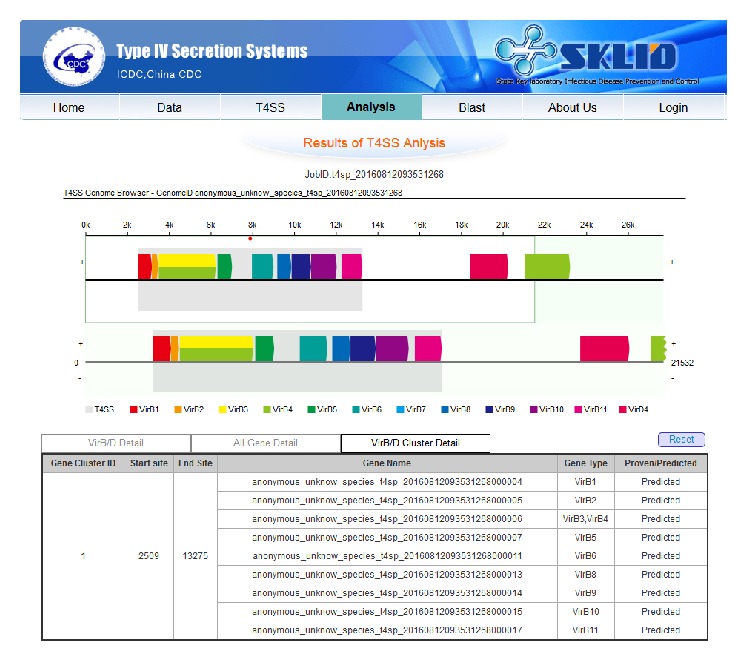
The web page covering a genome browser and tables about T4SS genes for the genome in the database or the sequence users submitted.

**Figure 5:**
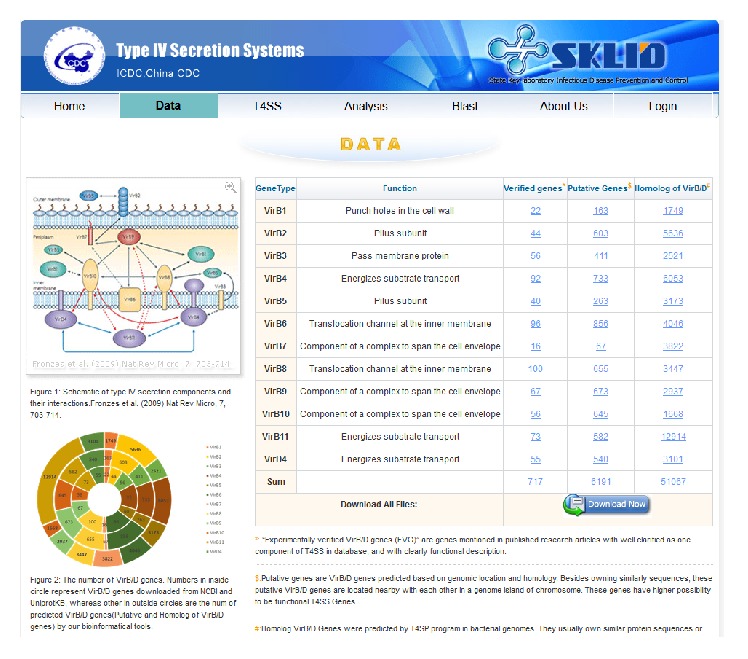
The data page.

**Figure 6:**
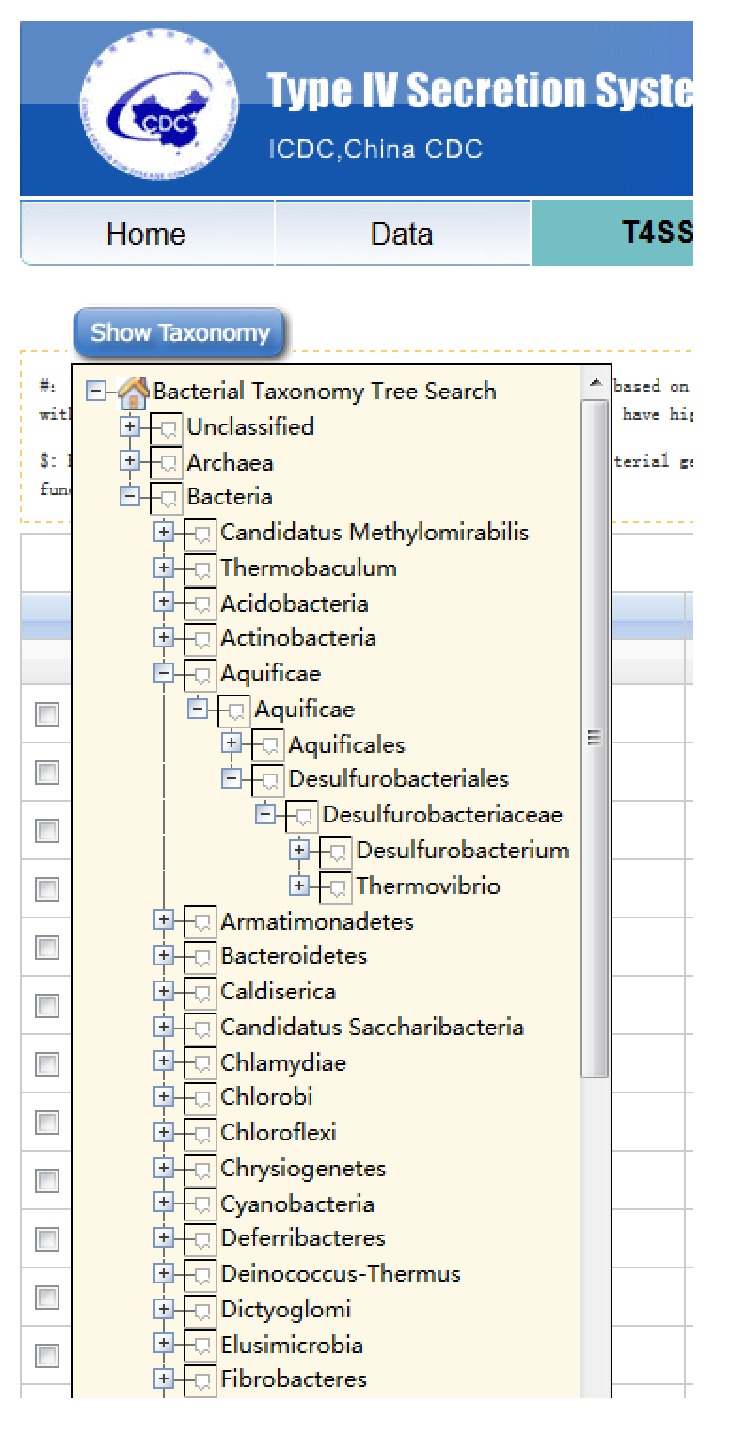
The taxonomy tree in 8 classic levels of bacteria (kingdom, phylum, class, order, family, genus, species, and strain).

In the new version of genome browser, all proven and predicted T4SS genes are shown in the figure using different color arrows, as well as T4SS gene cluster (red dot and grey shadow) (Figure 4). By clicking on the corresponding region, the gene information (name, type, location, direction, and sequence) would appear below. Furthermore, users could select their gene of interest by clicking on the gene in the gene browser and check the blast results of this gene by clicking “Blast (Proven)” or “Blast (Predict)” in the table. “Blast (Proven)” represents the blast function against T4SS genes, which have been proven by research, while “Blast (Predict)” represents the T4SS genes predicted by our bioinformatics tool. If users wish to know more detailed information about a gene, they could also click the “NCBI” button in the table located beneath the web page.

### 3.3. An Example of How to Use T4SP Database 2.0

Example data from* Streptococcus suis* (*S. suis*) are illustrated here to display the functions of our database.* S. suis* is a species of* Streptococcus* found in pigs and can cause human infections [11]. In our previous work, we reported the existence of candidate T4SS genes in* S. suis* for the first time and proved these function in the lethality of the virulent* S. suis* strain [6, 7]. In analysis page (Figure 1), we paste or upload the fasta format genome sequence of* S. suis* 98HAH33 and then click “Run T4SP.” The result page would be shown just as in Figure 4, which is a graphical report with genome browser and tables displaying all the information of T4SS genes related to this DNA sequence.

## 4. Conclusion

With the rapid increase in the number and diversity of sequenced microbial genomes, this web-based, user-friendly resource will continue to contribute to the investigation of T4SS genes and also provide insights into the potential pathogenic mechanisms of various microbial species. To our knowledge, T4SP 2.0 is the only dataset of T4SS database with online T4SS prediction analysis tools and blast web service. Our future goal for the T4SP database is to include more related genes, information, and bioinformatics analysis tools.

## Abbreviations

T4SS: Type IV secretion systems
T4SP: Type IV Secretion Project.

## Additional Points

*Availability and Requirements*. Project name is T4SP 2.0. Project home page is http://www.secretion.org/. Operating system(s) is platform independent. Programming language is Java. Other requirements are Java 1.3.1 or higher. License is GNU GPL. License is needed in case of any restrictions for use by nonacademics.
